# Combining transcranial direct current stimulation with a motor-cognitive task: the impact on dual-task walking costs in older adults

**DOI:** 10.1186/s12984-021-00826-2

**Published:** 2021-02-01

**Authors:** Nofar Schneider, Moria Dagan, Racheli Katz, Pablo Cornejo Thumm, Marina Brozgol, Nir Giladi, Brad Manor, Anat Mirelman, Jeffery M. Hausdorff

**Affiliations:** 1 Center for the Study of Movement, Cognition and Mobility, Neurological Institute, Tel Aviv Sourasky Medical Center, 6 Weizmann Street, Tel Aviv, Israel; 2 Sagol School of Neuroscience, Tel Aviv University, Tel Aviv, Israel; 3 Department of Physical Therapy, Sacker School of Medicine, Tel Aviv, Israel; 4 Department of Neurology, Sacker School of Medicine, Tel Aviv, Israel; 5 Hinda and Arthur Marcus Institute for Aging Research, Hebrew SeniorLife, Boston, MA USA; 6 Harvard Medical School, Boston, MA USA; 7 Division of Gerontology, Department of Medicine, Beth Israel Deaconess Medical Center, Harvard Medical School, Boston, MA USA; 8 Department of Orthopaedic Surgery, Rush Alzheimer’s Disease Center, Rush University Medical Center, Chicago, IL USA

**Keywords:** Gait, Dual-task, Neural stimulation, Cognitive, Dorsal-lateral pre-frontal cortex, tDCS, Aging, Fall risk, Accidental falls

## Abstract

**Background:**

The performance of a secondary task while walking increases motor-cognitive interference and exacerbates fall risk in older adults. Previous studies have demonstrated that transcranial direct current stimulation (tDCS) may improve certain types of dual-task performance, and, that tDCS delivered during the performance of a task may augment the benefits of stimulation, potentially reducing motor-cognitive interference. However, it is not yet known if combining multi-target tDCS with the simultaneous performance of a task related to the tDCS targets reduces or increases dual-task walking costs among older adults. The objectives of the present work were (1) To examine whether tDCS applied during the performance of a task that putatively utilizes the brain networks targeted by the neuro-stimulation reduces dual-task costs, and (2) to compare the immediate after-effects of tDCS applied during walking, during seated-rest, and during sham stimulation while walking, on dual-task walking costs in older adults. We also explored the impact on postural sway and other measures of cognitive function.

**Methods:**

A double-blind, ‘within-subject’ cross-over pilot study evaluated the effects of 20 min of anodal tDCS targeting both the primary motor cortex (M1) and the left dorsolateral prefrontal cortex (lDLPFC) in 25 healthy older adults (73.9 ± 5.2 years). Three stimulation conditions were assessed in three separate sessions: (1) tDCS while walking in a complex environment (tDCS + walking), (2) tDCS while seated (tDCS + seated), and (3) walking in a complex environment with sham tDCS (sham + walking). The complex walking condition utilized virtual reality to tax motor and cognitive abilities. During each session, usual-walking, dual-task walking, quiet standing sway, and cognitive function (e.g., Stroop test) were assessed before and immediately after stimulation. Dual-task costs to gait speed and other measures were computed.

**Results:**

The dual-task cost to gait speed was reduced after tDCS + walking (p = 0.004) as compared to baseline values. Neither tDCS + seated (p = 0.173) nor sham + walking (p = 0.826) influenced this outcome. Similar results were seen for other gait measures and for Stroop performance. Sway was not affected by tDCS.

**Conclusions:**

tDCS delivered during the performance of challenging walking decreased the dual-task cost to walking in older adults when they were tested just after stimulation. These results support the existence of a state-dependent impact of neuro-modulation that may set the stage for a more optimal neuro-rehabilitation.

*Trial registration:* Clinical Trials Gov Registrations Number: NCT02954328.

## Background

During many activities of daily living, walking is not performed alone. Instead, it is often completed under “dual-task” conditions, for example, walking while talking, while navigating through a complex environment, and/or while negotiating obstacles [1]. These challenging conditions require additional attention and cognitive resources, as compared to walking alone. The cognitive abilities involved in dual-tasking are mediated in part by neuronal activity in the left dorsolateral prefrontal cortex (lDLPFC), a brain region that plays a critical role in executive functions [2]. This reliance on cognitive function increases with age as the automaticity of motor function declines. At the same time, with aging, these cognitive functions are less able to fully compensate for the motor deficits that are common among older adults [3].

When a cognitive task is performed during walking, the two tasks compete for shared and limited cognitive resources [4]. In older adults, as compared to younger individuals, walking appears to require more cognitive input, potentially to compensate for age-associated alterations in the locomotor control system. [5–7] Competition of shared cognitive resources during dual-task walking thus leads to motor-cognitive interference and dual-task “costs” (i.e., decrements) to locomotor control and/or cognitive task performance [5, 6, 8–10]. Moreover, among older adults, those who exhibit greater dual-task costs are more likely to suffer falls and their often grave consequences in the future [11–13]. One way of potentially reducing these dual-task costs and the negative impact of motor-cognitive interference is to enhance its components, i.e., motor or cognitive function.

Transcranial direct current stimulation (tDCS) is a non-invasive, safe [14], and inexpensive means of selectively modulating cortical excitability [15]. Previous work has shown that anodal tDCS targeting the primary motor cortex (M1) during sitting improved motor function and gait patterns, as compared to sham stimulation in healthy young and older adults [16–19]. In addition, M1 stimulation enhanced implicit motor learning [20], improved balance control [17], and reduced dual-task costs [17–19]. tDCS studies have also examined the impact of targeting the lDLPFC, while seated and resting, on cognitive and/or motor functions. In healthy older adults, a single 20-min session of anodal tDCS targeting the lDLPFC increased the excitability of this region [21] and improved multiple aspects of executive functions [14, 18, 22, 23] Interestingly, tDCS targeting the lDLPFC while seated at rest also attenuated the dual-task costs and improved gait adaptation and postural control in healthy young and older adults [17–19]. Given the positive impact of M1 and lDLPFC stimulation separately, one might expect that multi-target tDCS designed to simultaneously facilitate the excitability of both of these regions [24] will have particularly beneficial effects on the motor-cognitive interference that typically occurs during dual-task walking in older adults. Although dual-task costs were not explicitly examined, findings from a study among patients with Parkinson’s disease [24] support this notion.

Recently, several studies have investigated the effects of combining tDCS administration with either motor or cognitive practice, suggesting that the stimulation impact is state dependent [25–31], at least in some patient populations and for certain motor and cognitive outcomes. Putatively, applying tDCS concurrently with the performance of a task that involves the same brain networks that are targeted by tDCS augments cortical excitability and neuroplasticity in the stimulated brain networks [25–28, 32]. It has been proposed that the impact of tDCS alone can be amplified when combined with a relevant task since the combination can generate synergy and maximize individual effects [32]. Still, while some studies suggest that beneficial effects are achieved when coupling these two methods [25, 26, 28], others emphasize the possibility of inducing detrimental effects [29, 30, 33]. One possible explanation for these divergent findings is the variations in the design and protocols examined, such as the site and parameters of stimulation, the nature of the task, and the study population [25–30, 32, 33]. Since none of the existing studies examined the impact of state-dependency on dual-task costs during walking in older adults, it is not yet clear if the combination of tDCS with activation of the neural substrate by a behavioral task yields a positive, negative, or no effect on the motor-cognitive interference that results from dual-task walking. Moreover, the effects of combining tDCS with the simultaneous performance of a related task have not been studied using multi-target stimulation.

To address these questions, in the present study, we examined the immediate after-effects of multi-target tDCS that was designed to simultaneously facilitate the excitability of the M1 and lDLPFC—delivered while walking in a complex environment (a task that putatively involves the same target brain regions)—on dual-task walking performance. We hypothesized that the reduction in the dual-task cost to gait speed (i.e., the difference between dual-task costs before versus after the intervention) would be different across the three conditions. We further hypothesized that the cost reduction after the multi-target tDCS delivered during the performance of a motor cognitive walking task (tDCS + walking) would be different than each of the other two conditions (tDCS + seated, and sham + walking), with the greatest reduction taking place in the tDCS + walking condition. Finally, we speculated that these changes would also be observed in other gait measures and in measures of standing postural sway and cognitive function.

## Methods

### Study design

**Fig. 1:**
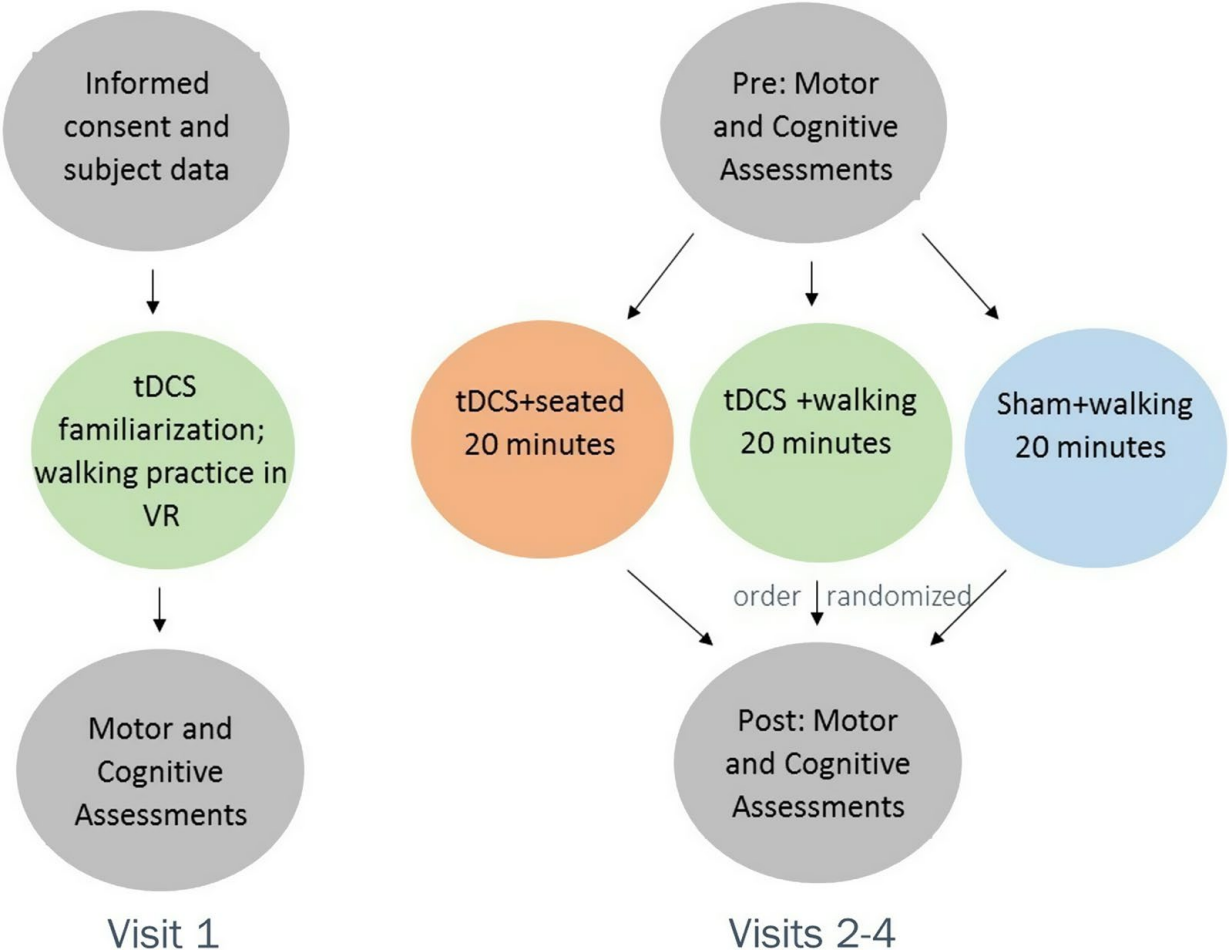
Overview of the study design. A cross-over, double-blind, randomized, sham-controlled tDCS pilot study, comparing three conditions. Visit 1 consisted of obtaining informed consent, familiarization with tDCS (the participants received some stimulation), and collection of subject data and demographics. During visit 1, subjects were also tested on all of the outcome measures that were used in subsequent visits to help to reduce any learning or practice effects. During each of the three subsequent evaluation sessions, gait, postural sway, and cognitive function were assessed before and after a single 20-min session of tDCS + walking, tDCS + seated, or sham + walking. Visit 2, 3, and 4 consisted of pre-test, intervention, and post-test. The intervention condition order was randomized across visits 2, 3, and 4. Each intervention session was separated by at least 3 days

As summarized in Fig. 1, a within-subject, cross-over, double-blind, randomized, sham-controlled anodal tDCS pilot study was conducted, comparing three conditions: (1) multi-target tDCS applied during the performance of motor-cognitive walking task (tDCS + walking); (2) real, multi-target tDCS applied while seated (tDCS + seated); and (3) sham stimulation applied during the performance of the motor-cognitive walking task (sham + walking). Each condition was separated by at least 3 days to minimize the likelihood that the stimulation effects of one session would carry over to another stimulation session [34]. During the tDCS while seated condition (tDCS + seated), the subjects were instructed to sit quietly and keep their eyes open. The order in which these three conditions were tested was randomized across participants. In addition, before the testing of these conditions, at the first study visit, all subjects were familiarized with tDCS and were tested on all of the outcome tests to minimize the impact of practice and learning in the subsequent visits. During each of the three subsequent experimental sessions, gait, postural sway, and cognitive function were assessed before and immediately after a single 20-min session of tDCS + walking, tDCS + seated, or sham + walking. All testing for an individual subject was typically carried out over 2 weeks.

### Participants

Subjects included healthy older men and women 65–85 years of age. The study was approved by the local ethics committee. All subjects provided informed written consent before their participation. Before enrolling in the study, all subjects completed a medical history questionnaire, and a safety and screening questionnaire to rule out contraindications to tDCS. Exclusion criteria included: (1) self-report of orthopedic, neurological (e.g., Parkinson’s disease, history of major stroke), uncontrolled diabetes, or psychiatric disorder, (2) history of seizures, and (3) any implanted metals in the head area. In addition, subjects were included if they could walk independently (without any assistance), scored 21 or higher on the Montreal Cognitive Assessment (MoCA) [35], and were not taking prescription medications likely to directly impact gait or cognitive function (e.g., psychiatric medications).

### Interventions

**Fig. 2:**
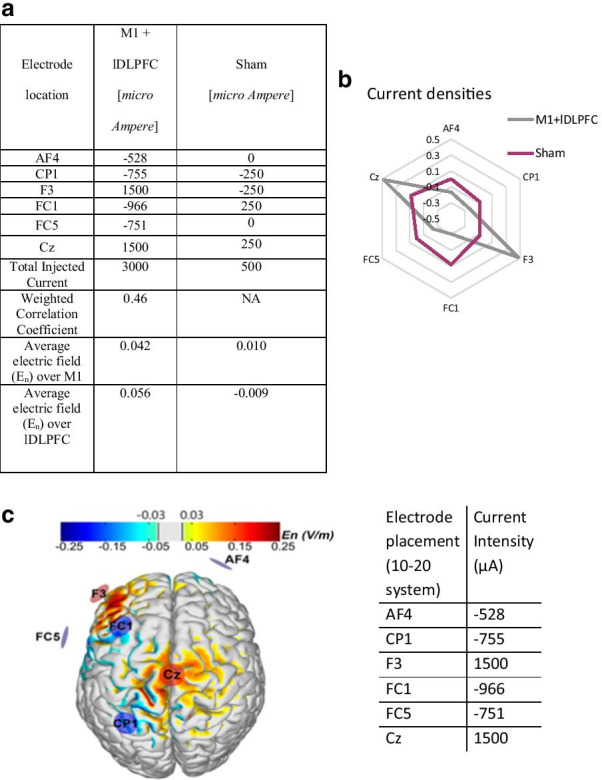
**a** Current intensities by locations of the electrodes, according to the 10–20 system, Total Injected Current, Weighted Correlation Coefficient, and average electric field (V/m) values are presented for each Montage. **b** Current densities (mA/cm^2^) and electrodes locations, according to the 10–20 system. Sham absolute densities were up to 0.08 mA/cm^2^. Electrodes FC5 and AF4 did not deliver current in the sham condition. **c** The targets of tDCS by montages targeting the left dorsolateral prefrontal cortex (L-DLPFC) and the primary motor cortex (M1). Modeling was carried out with the ‘Stimweaver’ algorithm on a standard brain to visualize the distribution of the component of the tDCS-induced electric field normal to the cortical surface. Warmer and cooler colors represent greater positive or negative normal component (En) of electrical field, respectively. Positive (current injecting, anodes) electrodes are in red; negative electrodes (cathodes) are in blue. The montage (electrode placement and current parameters) for each stimulation condition was developed using the Stimweaver® optimization technique on a realistic template head model. The stimulation regions-of-interest (ROIs) (i.e., the left DLPFC and M1) were determined via parcellation of Brodmann areas (i.e., L-DLPFC: BA 46; SM1: BA 1–4, within the leg area). The montage was designed to facilitate the excitability of the designated ROI, while at the same time optimally distributing the injected currents to minimize potential effects elsewhere in the brain. To do so, the Stimweaver algorithm was used to optimize the component of the electric field normal to the cortical surface (i.e., E_n_) over each ROI, as this component of the induced electric field polarizes pyramidal cells in the cortex and thus has been linked to the concurrent effects of stimulation on cortical excitability. The specific objective of each optimization was to minimize the (weighted) least-squares difference between the target E_n_-field distribution and the modeled E_n_ induced by the tDCS. The target E_n_-field was set to + 0.25 V/m over each designated ROI, and 0 V/m over the remaining regions

Anodal tDCS, in which a positive current is applied to facilitate depolarization, was applied for 20 min via an array of 6 Ag/AgCl electrodes (Pi-electrodes of π cm^2^ contact area), using the Starstim tDCS device and software (Neuroelectrics, Corp.). The ramp-up and ramp-down periods were 59 s each. The placement and current delivered through each electrode were optimized using the Neuroelectrics Stimweaver® Stimulation Optimization Service [24, 36] (see Fig. 2). The location of the electrodes was determined according to the 10–20 EEG placement system. To blind both the subject and the operator, the same electrode placement was used for all three conditions. For the tDCS + walking and tDCS + seated conditions, the tDCS montage was multi-target, targeting both lDLPFC and M1, as previously described [24]. The current was delivered during the entire 20-min sessions for both active conditions. In the sham stimulation condition, non-zero currents were applied (see Fig. 2). The system enables effective double-blinding of tDCS condition (real vs. sham) [24, 36]. In addition, blinding efficiency was monitored using a questionnaire. After each session of stimulation, subjects were asked to state if they believed they received real or sham stimulation, as well as their confidence level in this belief on a scale of minus three to three, with three reflecting greatest confidence, minus three reflecting lowest confidence, and zero indicating that the subject is not sure whether the stimulus was real or sham.

The tDCS device is small and portable, connected via Bluetooth to a computer, and its electrodes are connected to a head cap that fits each subject’s head size thus enabling both walking and tDCS stimulation simultaneously. Additionally, the tDCS software interface allowed us to make sure that the connection between the electrodes and scalp was maintained throughout the tDCS session and to continuously monitor the electrode impedance. Hence, we were able to ensure that the movement of the subjects while seated or during walking did not affect the connection of the electrodes.

### Walking apparatus

**Fig. 3:**
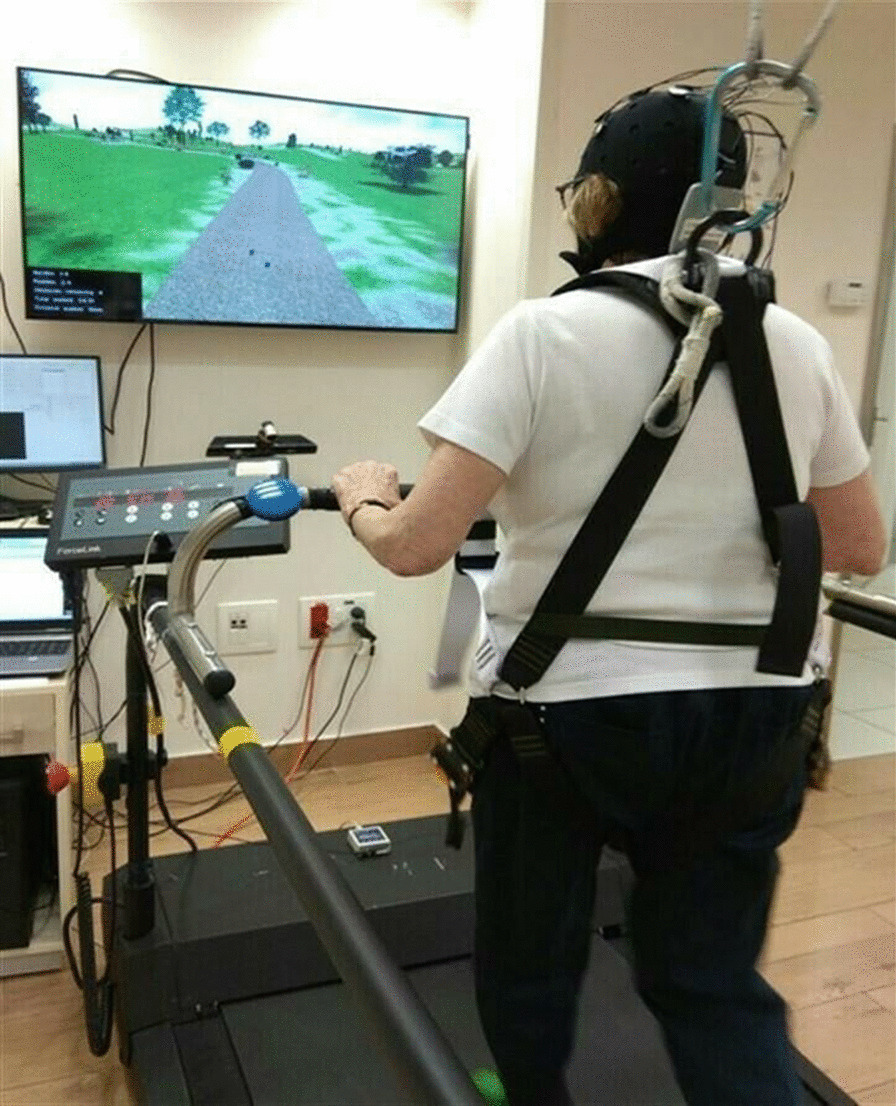
Picture of the tDCS during motor and cognitive practice. The practice is a computer-simulated non-immersive virtual reality (VR) treadmill training in which cognitive and motor aspects are both targeted [37]. The tDCS performed simultaneously targets both the M1 and lDLPFC. Subjects walked while wearing a safety harness to prevent falls during training

The motor-cognitive walking intervention condition included walking on a treadmill in a 2-dimensional virtual reality (VR) environment [37] (see Fig. 3). Briefly, the VR system consists of a motion-capture camera and a computer-generated simulation. The camera records the movement of the subject’s feet while they walk on the treadmill and projects it onto a large screen, where the subjects had to negotiate real-life challenges such as obstacles, multiple pathways, and distractions that require continuous adjustment of stepping [37]. The VR treadmill paradigm was designed to train obstacle negotiation strategies in a complex and enriched environment that requires the regulation and adaptation of gait while simultaneously engaging executive control, attention, and planning [38]. This task was chosen for our study since it involves the activation of the brain regions targeted by the tDCS (M1 and lDLPFC) [39]. A previous study in older adults showed that 6 weeks of VR treadmill training significantly improved gait, cognitive function, and fall incidence rates and that these changes were associated with changes in the activation of the pre-frontal cortex [37].

### Assessments

Gait and postural sway parameters were recorded using 3 wearable inertial measurement units (Opal, APDM, USA) and a 25-m instrumented walkway (Zeno mat, PKMAS software, USA). The gait assessment consisted of: (a) single-task, usual walking, (b) dual-task walking, (c) single-task quiet standing (30 s), (d) dual-task quiet standing (30 s). During the gait testing, subjects walked back and forth across the walkway (i.e., 50 m of walking). In the dual-task conditions, subjects were asked to walk or stand while serially subtracting three from a random 3-digit number provided immediately before each trial. Previous studies have shown that serial 3 subtractions lead to dual-task costs, i.e., decreased gait velocity and increased gait variability [5, 8–11, 40, 41]. Cognitive function was assessed by determining differences in the number of serial subtractions performed during seated single-task subtraction as compared to while walking. Also, two cognitive tests were performed, i.e., the Stroop Color and Word Test [42, 43] and the Symbol Digit Modalities Test (SDMT) [44], both require executive functions. The motor and cognitive assessments were done in the same order each visit.

The primary outcome was the dual-task cost to gait speed (i.e., percent decrement in performance between single-task and dual-task walking conditions) [8–10, 45] comparing baseline performance to post-intervention gait speed. The dual-task cost was calculated as follows:

$${\text{Cos}} t = \frac{{Speed_{DT} - Speed_{usual} }}{{Speed_{usual} }} \times 100$$

We used this parameter as our primary outcome since gait speed is a valid, reliable, sensitive measure of an older adult’s motor and cognitive abilities [46], and since the dual-task cost to gait speed has been related to multiple negative outcomes in older adults [6, 11, 12]. Gait speed was determined by the instrumented gait mat by dividing the length of the instrumented walkway by the time it took the subject to walk over it (and averaged across the multiple passes on the walkway). The secondary outcomes were the dual-task cost to (a) stride time, (b) double support time, (c) stride time variability (quantified as the coefficient of variation), (d) swing time variability (quantified as the coefficient of variation), and (e) step regularity, a measure of left–right asymmetry [47]. These measures were selected since they represent different aspects of gait [48, 49], which allowed us to examine whether any changes following interventions were specific to gait speed, or whether it affected multiple gait domains.

The dual-task cost to sway measures and the scores on the Stroop and the SDMT tests were explored to examine if the hypothesized effects of stimulation with walking were specific to gait (speed) or if they extended beyond gait. The following sway measures were used in the statistical analysis. Dual-task costs to sway frequency: the frequency (Hz) of the oscillations of the center of pressure calculated as the number of peaks in the anterior–posterior direction (i.e. changes in direction from forward to backward or vice versa) divided by the measurement time. Dual-task costs to sway path: the length (mm) of the trajectory of the center-of-pressure sway in the anterior–posterior direction. Dual-task costs to sway velocity: the average velocity (mm/sec) of the center-of-pressure in the anterior–posterior and medial–lateral directions.

The Stroop test assesses executive functions such as cognitive flexibility, vigilance, and divided attention, response inhibition, and other executive domains related to the lDLPFC cortex [42, 43]. In the first trial of this test, columns of names of colors (red, blue, black, and green) were printed in black ink (i.e., black condition). In the second trial, columns of names of colors were printed in red, blue, black, or green ink that matched the named color (i.e., congruent condition). In the third trial, the names of colors were printed in colored ink (red, blue, black, or green) that did not match the named color (i.e., interference condition). Our results are based on the scores obtained in the third trial and the most challenging condition since the cognitive dimensions tapped by this trial are the ones we expect to be affected by the intervention. The SDMT assesses neurocognitive functions such as attention control, information processing speed, and visual scanning [44]. The Stroop and SDMT results were calculated as—$\frac{{{\text{post}}\;{\text{intervention}}\;{\text{score}} - {\text{pre}}\;{\text{intervention}}\;{\text{score}}}}{{{\text{pre}}\;{\text{intervention}}\;{\text{score}}}}$.

The serial subtraction task assesses concentration, selective attention, working memory, and information processing speed, and since it’s performed during walking it also demands dual-tasking abilities. Measures of sway and gait were evaluated using previously described methods [50].

### Statistical analyses

Subject characteristics at baseline and all primary and secondary outcomes were summarized using descriptive statistics. Data were tested for normality and homogeneity of variance (Mauchly’s test of sphericity) and are summarized as mean and standard deviation. Extreme outliers (i.e., more than 3.0 times the interquartile range below the first quartile or above the third quartile) were transformed to the next highest / lowest (non-extreme outlier) value. We first examined our primary hypothesis that the reduction in the dual-task cost to gait speed (i.e., the difference between dual-task costs before versus after each individual intervention) would be different across the three conditions. We used repeated measures analysis of variance (RM ANOVA) with a sphericity-assumed correction for related conditions to compare the effects of each treatment condition. The level of statistical significance was set at p < 0.05. The dependent variable was cost reduction, defined as ${Cost}_{post}-{ Cost}_{pre}$ (where pre and post refer to the values measured with respect to that condition). In order to test our second hypothesis, this analysis was followed by post hoc paired-samples t-tests using Bonferroni corrections for multiple comparisons with adjusted alpha levels to evaluate pairwise comparisons among conditions. Similar analyses were applied to the secondary outcomes (e.g., the other measures of gait and cognitive test scores). Pearson’s correlation coefficients determined the strength of the linear relationship between two variables. Statistical analyses were conducted using the Statistical Package for the Social Sciences (SPSS) for Windows, Version 23.0 (IBM corp.).

## Results

### Subjects characteristics

Thirty subjects were recruited for this study; five subjects dropped out for reasons unrelated to the study, leaving twenty-five subjects who completed all parts of the study. Subject characteristics are summarized in Table 1. The tDCS treatment was well tolerated with no unexpected side effects reported. In addition, no technical faults were observed when the tDCS was performed during walking.

**Table 1:** Subject characteristics

	Mean ± SD	Range
Age (years)	73.9 ± 5.2	65.3–84.0
Gender (female/male)	20/5	–
Education (years)	15.3 ± 2.3	11–20
Body Mass Index (kg/m^2^)	26.6 ± 3.0	21.3–32.5
Montreal Cognitive Assessment (max 30)	26.6 ± 2.8	21–30
Single-task gait speed (cm/sec)^#^	113.1 ± 20.9	60.4–159.9
Dual-task gait speed (cm/sec)^##^	103.3 ± 22.7	50.3–146.9
Single-task stride time (ms)	1075 ± 91	880–1260
Single-task double support time (ms)	342 ± 75	230–820
Single-task step regularity (NU)	0.66 ± 0.11	0.39–0.82
Single-task stride time variability (%)	2.5 ± 0.9	1.1–3.65
Single-task swing time variability (%)	11.1 ± 16.8	1.94–56.7

^#^All gait values reported based on the baseline value of the first tDCS session

^##^Indicates a significant difference (p < 0.01) between the single-task and the dual-task gait speeds

### tDCS blinding efficacy

After sham + walking and after tDCS + walking the majority of subjects (72% after both conditions) reported that they received real stimulation. After tDCS + seated 56% of the subjects thought they received real stimulation. No significant difference (F(2,48) = 0.775, p = 0.466) was found in the confidence level score when comparing between the tDCS + walking (1.48 ± 1.56), the tDCS + seated (0.92 ± 1.71), and the sham + walking (1.28 ± 1.59) conditions.

### Effects on the dual-task cost to gait speed

**Fig. 4:**
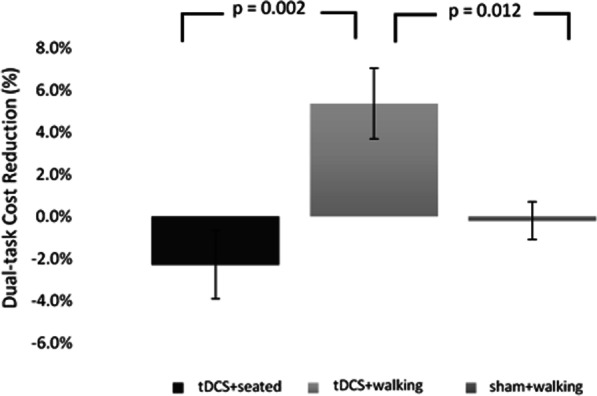
The cost reduction for gait speed following each tDCS condition. Repeated measures ANOVA comparing the three interventions revealed significant differences (F(2,48) = 7.576, p = 0.001) between the cost reduction of the three interventions (defined here as the change in dual-task costs before versus after the relevant intervention). Post hoc analysis with adjusted alpha levels of 0.025 per test revealed that the cost reduction in the tDCS + walking condition was significantly greater than the tDCS + seated condition and the sham + walking condition. The error bars represent the standard error. For this analysis, higher (positive) values reflect improved performance and lower costs

**Fig. 5:**
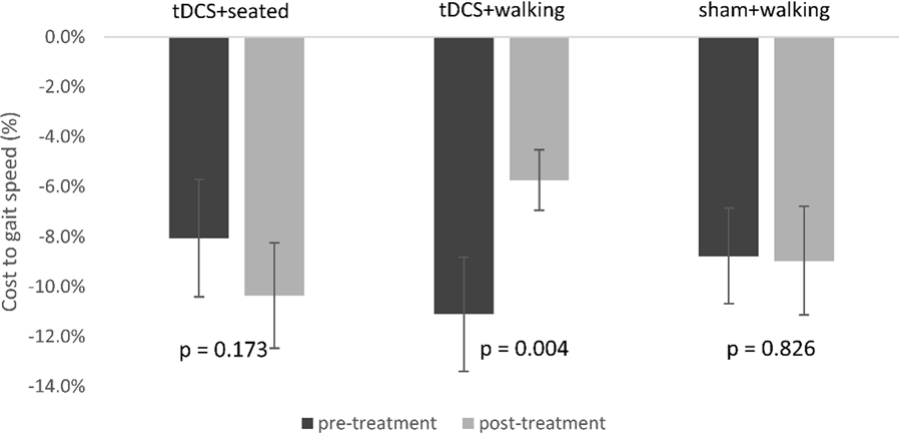
Post hoc, within condition analyses showing the effect of the dual-task costs on gait speed before and after each tDCS condition. In the tDCS + walking condition, the dual-task cost was significantly lower (p = 0.004) after the intervention as compared to before. In contrast, no statistically significant difference was observed between the dual-task costs before and after the tDCS + seated or before and after the sham + walking conditions

Baseline values (i.e., before the interventions) of the dual-task cost to gait speed were not different (F(2,48) = 1.967, p = 0.151) across the three stimulation sessions. Repeated measure ANOVA indicated that the dual-task cost reduction to gait speed (change from pre-to-post cost) was dependent upon stimulation condition (F(2, 48) = 7.576, p = 0.001). The tDCS + walking condition had a significantly higher (i.e., better) cost reduction compared to both tDCS + seated and sham + walking (see Fig. 4). Consistent with this finding, as shown in Fig. 5, post hoc analyses revealed that the dual-task cost to gait speed was significantly reduced, by almost 50% on average, after tDCS + walkng. In contrast, in both the tDCS + seated condition and in the sham + walking condition, the pre-stimulation and the post-stimulation costs were similar, i.e., unchanged in response to the stimulation. Pearson correlations showed that the dual-task cost and the cost reduction values in each of the three conditions were not associated (p > 0.47) with age, gender, body-mass-index, or MoCA scores.

Dual-task gait speed was significantly faster (t(24) = − 2.136, p = 0.04, paired t-test) after the tDCS + walking (108.1 ± 20.3 cm/s) as compared to before (102.9 ± 24.6 cm/s). However, there was no significant difference (t(24) = 0.543, p = 0.592) between the usual gait speeds before (115.3 ± 21.1 cm/s) and after (114.4 ± 18.6 cm/s) the tDCS + walking condition (result of paired t-test). No significant differences were found before and after the tDCS + seated in the dual-task gait speed (t(24) =  − 0.211, p = 0.835) and single-task gait speed (t(24) = 1.722, p = 0.098), and before and after the sham + walking in the dual-task gait speed (t(24) = 0.191, p = 0.85) and single-task gait speed (t(24) = 0.322, p = 0.75) (results of paired t-tests).

### Effects on other gait measures

The dual-task costs of other gait measures were significantly reduced (p < 0.02) following the tDCS + walking condition, as compared to the costs before the intervention, as shown in Table 2. For many of these measures, the cost reduction after the tDCS + walking condition was significantly reduced as compared to the tDCS + seated and the sham + walking conditions (p < 0.024; see Table 3).

**Table 2:** The dual-task costs to gait measures, before and after the tDCS + walking condition

Gait measure	Pre-cost	Post-cost	t (24)	P-value
Stride time	− 7.5 ± 10.3%	− 2.9 ± 5.7%	1.42	0.013
Double support time	− 11.9 ± 13.7%	− 7.6 ± 7.9%	1.06	0.016
Step regularity	− 10.1 ± 11.4%	− 2.8 ± 12.9%	− 1.95	0.006
Stride time variability	− 193.2 ± 59.4%	− 49.2 ± 37.3%	2.61	0.020
Swing time variability	− 77.0 ± 86.3%	− 18.0 ± 47.7%	2.21	0.009

The dual-task costs following the tDCS + walking condition were significantly reduced (i.e., improved) as compared to the costs before the intervention. In this analysis, lower costs are better

**Table 3:** The effects of tDCS condition on the dual-task cost to gait measures

Cost reduction tDCS + seated	Gait measure	Cost reduction tDCS + walking	Cost reduction sham + walking	RM ANOVA P-value	F_(2,48)_	P-value (tDCS + seated vs. tDCS + walking)	P-value (sham + walking vs. tDCS + walking)
− 0.2 ± 6.0%	Stride time	3.2 ± 3.9%	− 0.7 ± 2.7%	**0.005**	6.010	0.008*	0.002*
− 0.6 ± 8.5%	Double support time	5.2 ± 10.4%	− 0.2 ± 6.3%	**0.03**	3.768	0.03	0.04
− 2.8 ± 12.0%	Step regularity	7.3 ± 11.9%	2.3 ± 9.1%	**0.015**	4.579	0.019*	0.024*
38.6 ± 35.5%	Stride time variability	143.9 ± 57.8%	− 60.2 ± 40.2%	**0.013**	4.765	0.155	0.019*
6.0 ± 106.8%	Swing time variability	58.9 ± 104.1%	− 38.5 ± 115.6%	**0.012**	4.812	0.131	0.013*

In this analysis, higher values reflect a larger and better cost reduction

Bolded p-values values indicate significant differences across conditions

Post hoc analysis revealed between which conditions the cost reduction differed statistically significantly. *Values that are lower than the alpha levels after the Bonferroni correction

### Effects on the dual-task costs to postural sway measures

No significant differences were found between the values before and after the tDCS + walking in the dual-task costs to sway frequency (t(24) = − 0.37, p = 0.712), sway path length (t(24) = − 0.714, p = 0.482) and sway velocity (t(24) = 1.12, p = 0.563). The single-task values of these three sway measures (frequency, path length and velocity) did not change (t(24) = 0.52, p = 0.608; t(24) = 0.177, p = 0.861; t(24) = 0.821, p = 0.42; respectively). These measures of sway also did not improve after the tDCS + seated (t(24) = 0.776, p = 0.446; t(24) = 0.073, p = 0.943; t(24) = -0.96, p = 0.346) and after the sham + walking (t(24) = 1.073, p = 0.294; t(24) = 0.211, p = 0.834; t(24) = − 0.176, p = 0.862).

### Effects on cognitive function

RM ANOVA showed that the change in Stroop performance before and after each stimulation differed (F(2,48) = 6.019, p < 0.005). Post hoc analyses showed that after the tDCS + walking condition, the number of words that were correctly read in the interference condition of the Stroop test (42.4 ± 8.8) was significantly higher (better) (t(24) = − 4.2, p < 0.001) than the number of words that were read before stimulation (37.4 ± 8.1). No significant difference was observed when comparing the pre-stimulation (36.3 ± 7.3) with the post-stimulation (37.8 ± 8.8) in the tDCS + seated condition, or in the sham + walking condition (from: 38.3 ± 8.4 to: 38.0 ± 9.9). Baseline values (i.e., before intervention) of the cognitive tests in the tDCS + walking was not different (p > 0.30) from that of the baseline values in the other two stimulation sessions. For the SDMT, the pre-stimulation score (43.2 ± 8.1) did not differ significantly (t(24) = − 0.8, p = 0.415) from the post-stimulation score (44.2 ± 8.3) in response to the tDCS + walking condition. Moreover, when comparing the improvement values of the three conditions, no significant differences were observed (F(2,48) = 0.006, p = 0.994). In the serial subtraction task, there was a trend toward an improvement (t(24) = − 1.9, p = 0.069) in the score after the combined condition (21.6 ± 7.3) as compared to its value before (20.1 ± 6.8). However, the improvements in scores on the serial subtraction task were not statistically significant between the three conditions (F(2,48) = 0.519, *P* = 0.599). No correlation was found (p > 0.199) between the number of correct answers and the dual-task gait speed after the tDCS + walking (r = -0.331, p = 0.107), tDCS + seated(r = − 0.281, p = 0.173) and sham + walking(r = − 0.217, p = 0.298) conditions.

## Discussion

In this within-subject, cross-over study, a single-session of multi-target tDCS that was designed to facilitate both M1 and the lDLPFC during the performance of a complex walking task reduced the negative impact of dual-tasking on gait when tested immediately after stimulation. The positive effects of the combined condition (tDCS + walking) on dual-task costs were reflected in gait speed and in measures of gait variability, regularity, rhythm, and double support. In contrast, walking with sham stimulation (sham + walking) or tDCS without walking (tDCS + seated) did not cause changes in dual-tasking or cognitive performance. No differences between the three conditions were observed in the number of subjects who believed they received real tDCS or in the confidence levels about their guesses. In addition, performance on the serial subtraction task was similar across all conditions and there was no significant association between the number of correct responses on the serial subtraction test and dual-task gait speed, suggesting that the improvement in dual-tasks costs to gait were not driven by changes in the performance of the serial subtraction task. Together, these results suggest that the observed impact of the combined condition of real tDCS and walking was not simply due to a placebo effect, was not due to the effects of walking in the cognitively demanding condition by itself, and was not due to familiarization with the outcome assessments. The present results thus support the idea of a state-dependent impact of multi-target tDCS on dual-tasking walking. tDCS may be more effective at reducing dual-task costs if it is administered during a cognitively-challenging walking task, as opposed to during seated rest.

One possible interpretation of these results is that the stimulation combined with a challenging walking condition led to enhanced cortical excitability immediately after the intervention that in turn led to a decrease in motor-cognitive interference, as reflected in the dual-task cost reduction. In contrast to some reports that found that task performance harmed stimulation efficacy [27, 29, 30, 33], this possibility is consistent with several studies that demonstrated that, for other outcomes and in other cohorts, the combination of task performance and tDCS can enhance tDCS efficacy [25, 26, 28]. Here we showed, for the first time, that the simultaneous performance of walking in a cognitively demanding environment during tDCS enhances the impact of the tDCS on dual-task gait costs in older adults.

In addition to mitigation of the dual-task cost to gait speed, tDCS delivered during walking improved several other aspects of dual-task gait, as well as a measure of executive function, when tested immediately after stimulation. The improvements on the Stroop test were only observed in the interference condition, the most cognitively challenging condition (data not shown for the other Stroop conditions). This is consistent with previous studies [51–53] that have shown an increased score and better performance on the interference condition than on the congruent condition, which implies the positive impact of prefrontal tDCS on executive functions. This positive effect of tDCS is similar to other studies that have seen improvements in executive function after targeting the pre-frontal cortex [24, 54]. Moreover, usual-walking gait speed did not improve, and standing sway and other aspects of cognitive function did not benefit from this combination. One parsimonious explanation for these positive and negative findings is that improvements were observed in outcome measures, both cognitive and motor, that more closely resemble the challenging walking condition. On the other hand, not all motor and cognitive processes improved after the combined condition, but only those that were targeted by the combined practice and tDCS. These findings are consistent with the notion [25, 26, 28] that the positive after-effects of tDCS on performance within a given task tend to be greater if that task closely resembles the task completed during tDCS administration.

These findings combined with the observed greater impact of tDCS + walking over tDCS alone and walking alone can be explained by several possible neural mechanisms. Long Term Potentiation (LTP) induction [55] and the glutamatergic system modifications [32] have a significant role in learning and memory processes that may occur during both tDCS and performance of the task [56, 57]. Moreover, voltage-gated sodium channels may open as a response to tDCS and the performance of the behavioral task [58, 59]. Since these channels are essential for producing changes in the membrane potential, this may be another mechanism by which the combination of tDCS during task performance may enhance cortical excitability. The Hebbian-like activation theory [32] may also explain the present findings. According to this theory, the simultaneous activation of neurons by both tDCS and the performance of the task leads to pronounced increases in the synaptic strength (i.e., the voltage excursion produced in the postsynaptic neuron by an action potential in the presynaptic neuron) between the activated neurons [25]. Another possibility is that whereas tDCS facilitates excitability in a bulk of neural networks in a relatively nonspecific way, the simultaneous performance of the walking task activates specific neural networks. Thereby, the walking task causes these networks to be more sensitive and receptive to the tDCS [60], focusing and narrowing its impact. Moreover, the neurotransmitter dopamine induces a non-linear, dosage-dependent effect on the plasticity induced by tDCS [56, 57], which may also underlie the putative efficiency obtained from the combination. When comparing the tDCS during walking to tDCS while seated, it is also helpful to keep in mind possible non-neurological contributions to the observed differences (e.g., active muscles vs. sedentary muscles) In the future, it would be interesting to identify which of these mechanisms were at play here. Nonetheless, given the specificity of the findings of greater change in the combined condition compared to the single conditions, it can be assumed that at least one of these mechanisms (and possibly more than one) led to the enhanced effects of the combined stimulation and task performance.

Somewhat surprisingly, in contrast to several previous reports [17–20, 22, 23], the stimulation while seated condition did not reduce the dual-task costs in the present study. This disparity may be explained by several factors. The subject characteristics may impact the tDCS outcomes; a previous review [61] suggests that there is no clear evidence of beneficial effects from a single tDCS session in healthy adults. Moreover, the location of the electrodes might affect tDCS efficiency. While in most previous studies [17–20, 22, 23], tDCS targeting either the DLPFC [14, 17–19, 22, 23] or M1 [20] was delivered via large sponge electrodes that create diffuse electric fields that vary considerably across participants. The montage we utilized was relatively novel [24], targeting both the lDLPFC and M1 simultaneously (i.e., multi-target) using relatively small electrodes and current mapping to focus the stimulation in an attempt to enhance both cognitive and motor functions, without stimulating other regions. These aforementioned factors may explain why the stimulation while seated condition did not yield a positive effect on performance in the present study.

The present study has several limitations. We examined only the acute, immediate effects of a single exposure to tDCS. An important question that we did not address is how long the benefits of the combined stimulation with task performance endure. Previous work showed that 10 repeated stimulation sessions may be sufficient to lead to long-lasting retention effects for some outcomes [16, 62]. It remains to be seen if this would be the case for dual-task gait speed costs and whether long-term application not only improves gait and cognitive function but also leads to a reduction in fall risk and perhaps help to enhance or prevent the decline of executive function. In the future, it may also be interesting to see which specific aspects of the cognitively challenging walk are needed to enhance the impact of the stimulation and also to test whether the combination of tDCS and a task can benefit neurological populations. In addition, it would be interesting to investigate the mechanisms that contributed to the observed behavioral changes using excitability and imaging probes. Moreover, in the present study, we examined the impact of the combined condition with one tDCS montage that combined M1 and lDLPFC. It may, in the future, be informative to study if and how the combination of tDCS with the walking tasks depends on the specifics of the montage (e.g., to compare to M1 only or lDLPFC only stimulation). On that note, to more fully complete the picture, it would also be interesting to see if walking alone—without the addition of the virtual reality or some other cognitively challenging component—provides similar benefits to the tDCS as walking in the virtual reality environment. In the present study, we found that walking in that environment with the sham tDCS did not improve the dual-task costs, however, we did not examine if the performance in the VR improved.

We also note that the gender distribution was imbalanced; more women participated than men. Thus, differences in anatomy may have led to gender-related differences in impedance and modified the effects of the tDCS. In exploratory analyses, we confirmed that the main findings regarding the positive effects of the combined condition (tDCS + walking) on the dual-task cost to gait were not related to gender by adding sex as a covariate (its role was not significant, p = 0.513) and by examining each gender alone (when comparing pre-cost of gait speed to post-cost in this condition, the effect was significant in the women, p = 0.025, with a strong trend for the men, p = 0.053, who were a much smaller group). Still, in the future, it would be helpful to confirm that these apply similarly to both older adult men and older adult women. Finally, we examined one aspect of state-dependency, i.e., is tDCS more effective when it is delivered during the performance of a related task. As noted in the Introduction, other options may also take advantage of state dependency to enhance stimulation efficacy, e.g., by first delivering the tDCS and then carrying out the task or vice versa. The present findings set the stage for future work that is needed to investigate these possibilities in the context of dual-task walking in old adults.

## Conclusions

The study demonstrates the feasibility of delivering tDCS during walking among older adults. Moreover, the present findings support the notion that the simultaneous modulation of shared neural networks by tDCS and the performance of a related behavioral task can generate a synergistic effect whereby the resultant impact is greater than the impact imparted by either of the two components separately. This idea can, potentially, be applied in clinical research aimed at examining whether other tDCS-based protocols that focus on different brain regions and other outcomes can maximize the efficiency of tDCS when combined with an appropriate task. To optimize the effect of this kind of treatment protocol, one should keep in mind that the task performed during the tDCS should involve the same or similar neural network(s) that are activated by the neuro-stimulation. The present findings also shed light on possible factors that contribute to tDCS-induced brain plasticity, support the idea of state-dependence, and may help in the development of optimal tDCS-based therapeutic protocols designed to reduce the dual-task impact on gait and fall risk.

## Data Availability

The data will be made available from the authors based on reasonable requests.

## Abbreviations

tDCS: Transcranial direct current stimulation
lDLPFC: Left dorsolateral prefrontal cortex
M1: Primary motor cortex
